# Supplementary material for: A systems-biology approach to molecular machines: Exploration of alternative transporter mechanisms
Source: PLoS Comput Biol. 2020 Jul 2;16(7):e1007884. doi: 10.1371/journal.pcbi.1007884 (PMC7331975; doi:10.1371/journal.pcbi.1007884)
Supplement: S1 Text — Details regarding the string-based creation of states, state definitions, equivalent states/transitions, tempering procedures, and flux calculations. (PDF) [file pcbi.1007884.s001.pdf]

1 Detailed methods

1.1 String based state-space specification

The state string consists of a set of user-defined ‘base state’ strings, each of which describes a different characteristic of the machine. For a transporter, this includes the conformational state (inward or outward facing; IF or OF), sodium-ion state (bound to protein, intracellular/“inside”, or extracellular/“outside”; Nb, Ni, or No), and a substrate binding state (Sb, Si, or So), resulting in a 3D state-space. The inside and outside designations are necessary to define the direction of a transition (e.g., N binding from outside) but note that under the steady-state conditions analyzed here, species concentrations are held fixed but with differing values inside and outside. That is, transitions do not change the steady-state concentrations.

To examine transport mechanisms in heterogeneous environments, we add an additional binding base state for the competing substrate (W) following the same conventions (Wb, Wi, or Wo). These states are defined in an analogous manner: e.g., OF-Nb-Wi. By choice, S and W cannot both be bound simultaneously.

Ultimately, steady-state populations are calculated for distinguishable states. For example, the “OF-Nb-Si” and “OF-Nb-So” states are not distinguishable in steady state because inside and outside substrate concentrations are held fixed. (As noted above, the inside and outside designations are necessary to assign directionality to binding events.) In contrast, the “OF-Nb-Si” and “OF-Ni-Sb” states differ in their binding state making them distinguishable. Likewise the “OF-Nb-Si” and “IF-Nb-Si” states are distinguishable. Hence, as detailed below, the full set of states is mapped to a smaller set of physically unique states for determining populations. Furthermore, any set of indistinguishable states must be energetically equivalent with the same state energy, E_i, value. These indistinguishable states are “tied” together for the Monte Carlo procedure. Note that state populations cannot be directly inferred from the equilibrium E_i values because we are studying driven, non-equilibrium steady states.

1.2 State name definitions

OF	Outward-facing conformation
IF	Inward-facing conformation
No	Extracellular sodium ion
Ni	Intracellular sodium ion
Nb	Bound sodium ion
So	Extracellular substrate
Si	Intracellular substrate
Sb	Bound substrate
Wo	Extracellular decoy substrate
Wi	Intracellular decoy substrate
Wb	Bound decoy substrate

1.3 Equivalent states and transitions

We have defined groups of states that are physically equivalent at steady state with fixed extracellular and intracellular concentrations. After a species (e.g. ion or substrate) is transported, the physical state will remain the same because of the steady-state assumption. As an example, consider a hypothetical transporter of substrate (S) driven by a sodium ion (N). The state 'OF-Nb-So' describes the extracellular (outward) facing conformation (OF) with sodium-bound (Nb) and substrate unbound and in the extracellular region (So). This is physically equivalent to the state 'OF-Nb-Si', which only differs by the "location" of the unbound substrate (Si, substrate inside the cell). The location of a substrate or ion is needed in order to fully identify transitions – i.e., the origin (inside or outside) of substrate or ion in a binding process.

Transitions are similarly grouped based on states that are equivalent under the conditions stated above. Considering the same hypothetical transporter: an extracellular-to-bound sodium transition (No $\rightarrow$ Nb) would be physically equivalent for extracellular (So) and intracellular (Si) substrate in the outward-facing conformation (OF). The equivalent state and transition groups are constrained to share the same state or transition energy during the Monte Carlo (MC) energy perturbations for self-consistency.

To investigate the Hopfield kinetic proofreading model of transport, we have an additional constraint that groups 'equivalent' transitions for the substrate and decoy substrate. As an example, an inward-to-outward facing conformational transition with only the decoy bound (e.g. OF-No-So-Wb to IF-No-So-Wb) would be equivalent to an inward-to-outward facing conformational transition with only the substrate bound (e.g. OF-No-Sb-Wo to IF-No-Sb-Wo). This extra constraint prohibits a difference in transition energies between equivalent substrate and decoy transitions; effectively removing "internal proofreading" models from our search.

1.4 List of equivalent states/transitions for a cotransporter without decoy substrate

Equivalent states:

- IF-Ni-So, IF-No-Si, IF-No-So, IF-Ni-Si
- OF-No-So, OF-Ni-Si, OF-No-Si, OF-Ni-So
- IF-Ni-Sb, IF-No-Sb
- IF-Nb-So, IF-Nb-Si
- OF-No-Sb, OF-Ni-Sb
- OF-Nb-So, OF-Nb-Si

Equivalent transitions:

- OF-Ni-Sb⟷OF-Ni-Si, OF-No-Sb⟷OF-No-Si
- OF-Ni-So⟷OF-Ni-Sb, OF-No-So⟷OF-No-Sb
- IF-Nb-Si⟷IF-Ni-Si, IF-Nb-So⟷IF-Ni-So
- OF-No-So⟷OF-Nb-So, OF-No-Si⟷OF-Nb-Si
- OF-No-So⟷IF-No-So, OF-Ni-Si⟷IF-Ni-Si, OF-Ni-So⟷IF-Ni-So, OF-No-Si⟷IF-No-Si
- OF-Ni-Sb⟷IF-Ni-Sb, OF-No-Sb⟷IF-No-Sb
- IF-No-So⟷IF-Nb-So, IF-No-Si⟷IF-Nb-Si,
- OF-Nb-Si⟷OF-Ni-Si, OF-Nb-So⟷OF-Ni-So,
- OF-Nb-So⟷IF-Nb-So, OF-Nb-Si⟷IF-Nb-Si
- IF-No-So⟷IF-No-Sb, IF-Ni-So⟷IF-Ni-Sb,
- IF-No-Sb⟷IF-No-Si, IF-Ni-Sb⟷IF-Ni-Si

1.5 List of equivalent states/transitions for a cotransporter with decoy substrate

Equivalent states:

- OF-Nb-So-Wb, OF-Nb-Sb-Wo, OF-Nb-Sb-Wi, OF-Nb-Si-Wb
- IF-Ni-So-Wb, IF-No-Sb-Wo, IF-Ni-Sb-Wo, IF-No-Sb-Wi, IF-Ni-Si-Wb, IF-No-Si-Wb, IF-Ni-Sb-Wi, IF-No-So-Wb
- OF-No-So-Wb, OF-No-Si-Wb, OF-Ni-So-Wb, OF-Ni-Sb-Wo, OF-No-Sb-Wo, OF-Ni-Si-Wb, OF-Ni-Sb-Wi, OF-No-Sb-Wi
- IF-Nb-So-Wo, IF-Nb-Si-Wo, IF-Nb-Si-Wi, IF-Nb-So-Wi
- IF-Ni-So-Wo, IF-Ni-Si-Wo, IF-No-Si-Wi, IF-No-So-Wo, IF-No-Si-Wo, IF-Ni-So-Wo, IF-Ni-So-Wi, IF-No-So-Wi, IF-Ni-Si-Wi
- IF-Nb-So-Wb, IF-Nb-Sb-Wi, IF-Nb-Si-Wb, IF-Nb-Sb-Wo
- OF-Nb-So-Wo, OF-Nb-Si-Wo, OF-Nb-Si-Wi, OF-Nb-So-Wi
- OF-No-So-Wo, OF-Ni-So-Wi, OF-No-Si-Wi, OF-Ni-Si-Wo, OF-Ni-Si-Wi, OF-No-Si-Wo, OF-No-So-Wi, OF-Ni-So-Wo

Equivalent transitions:

- IF-No-So-Wb⟷IF-Nb-So-Wb, IF-No-Sb-Wi⟷IF-Nb-Sb-Wi, IF-No-Sb-Wo⟷IF-Nb-Sb-Wo, IF-No-Si-Wb⟷IF-Nb-Si-Wb
- OF-Nb-So-Wi⟷IF-Nb-So-Wi, OF-Nb-Si-Wo⟷IF-Nb-Si-Wo, OF-Nb-Si-Wi⟷IF-Nb-Si-Wi, OF-Nb-So-Wo⟷IF-Nb-So-Wo
- IF-Nb-So-Wb⟷IF-Nb-So-Wi, IF-Nb-Si-Wb⟷IF-Nb-Si-Wi, IF-Nb-Sb-Wo⟷IF-Nb-Si-Wo, IF-Nb-Sb-Wi⟷IF-Nb-Si-Wi
- OF-Nb-Si-Wb⟷IF-Nb-Si-Wb, OF-Nb-So-Wb⟷IF-Nb-So-Wb, OF-Nb-Sb-Wo⟷IF-Nb-Sb-Wo, OF-Nb-Sb-Wi⟷IF-Nb-Sb-Wi
- IF-Nb-Sb-Wi⟷IF-Ni-Sb-Wi, IF-Nb-So-Wb⟷IF-Ni-So-Wb, IF-Nb-Si-Wb⟷IF-Ni-Si-Wb, IF-Nb-Sb-Wo⟷IF-Ni-Sb-Wo
- OF-No-Sb-Wo⟷OF-No-Si-Wo, OF-No-Sb-Wi⟷OF-No-Si-Wi, OF-Ni-Si-Wb⟷OF-Ni-Si-Wi, OF-No-Si-Wb⟷OF-No-Si-Wi, OF-Ni-Sb-Wo⟷OF-Ni-Si-Wo, OF-Ni-So-Wb⟷OF-Ni-So-Wi, OF-No-So-Wb⟷OF-No-So-Wi, OF-Ni-Sb-Wi⟷OF-Ni-Si-Wi
- OF-Ni-Sb-Wo⟷IF-Ni-Sb-Wo, OF-Ni-So-Wb⟷IF-Ni-So-Wb, OF-No-Sb-Wi⟷IF-No-Sb-Wi, OF-Ni-Sb-Wi⟷IF-Ni-Sb-Wi, OF-No-Sb-Wo⟷IF-No-Sb-Wo, OF-Ni-Si-Wb⟷IF-Ni-Si-Wb, OF-No-Si-Wb⟷IF-No-Si-Wb, OF-No-So-Wb⟷IF-No-So-Wb
- IF-No-Si-Wb⟷IF-No-Si-Wi, IF-Ni-Sb-Wo⟷IF-Ni-Si-Wo, IF-No-So-Wb⟷IF-No-So-Wi, IF-No-Sb-Wi⟷IF-No-Si-Wi, IF-Ni-So-Wb⟷IF-Ni-So-Wi, IF-Ni-Sb-Wi⟷IF-Ni-Si-Wi, IF-No-Sb-Wo⟷IF-No-Si-Wo, IF-Ni-Si-Wb⟷IF-Ni-Si-Wi
- OF-Ni-Si-Wi⟷IF-Ni-Si-Wi, OF-No-So-Wi⟷IF-No-So-Wi, OF-Ni-Si-Wo⟷IF-Ni-Si-Wo, OF-No-So-Wo⟷IF-No-So-Wo, OF-Ni-So-Wo⟷IF-Ni-So-Wo, OF-No-Si-Wo⟷IF-No-Si-Wo, OF-No-Si-Wi⟷IF-No-Si-Wi, OF-Ni-So-Wi⟷IF-Ni-So-Wi

- OF-Nb-Sb-Wo⟷OF-Nb-Si-Wo, OF-Nb-So-Wb⟷OF-Nb-So-Wi, OF-Nb-Sb-Wi⟷OF-Nb-Si-Wi, OF-Nb-Si-Wb⟷OF-Nb-Si-Wi
- IF-No-Si-Wi⟷IF-Nb-Si-Wi, IF-No-Si-Wo⟷IF-Nb-Si-Wo, IF-No-So-Wi⟷IF-Nb-So-Wi, IF-No-So-Wo⟷IF-Nb-So-Wo
- OF-Nb-So-Wi⟷OF-Nb-Sb-Wi, OF-Nb-Si-Wo⟷OF-Nb-Si-Wb, OF-Nb-So-Wo⟷OF-Nb-So-Wb, OF-Nb-So-Wo⟷OF-Nb-Sb-Wo
- IF-Nb-So-Wo⟷IF-Ni-So-Wo, IF-Nb-Si-Wi⟷IF-Ni-Si-Wi, IF-Nb-Si-Wo⟷IF-Ni-Si-Wo, IF-Nb-So-Wi⟷IF-Ni-So-Wi
- OF-No-Si-Wi⟷OF-Nb-Si-Wi, OF-No-Si-Wo⟷OF-Nb-Si-Wo, OF-No-So-Wi⟷OF-Nb-So-Wi, OF-No-So-Wo⟷OF-Nb-So-Wo
- OF-Nb-Si-Wb⟷OF-Ni-Si-Wb, OF-Nb-So-Wb⟷OF-Ni-So-Wb, OF-Nb-Sb-Wo⟷OF-Ni-Sb-Wo, OF-Nb-Sb-Wi⟷OF-Ni-Sb-Wi
- OF-No-Sb-Wo⟷OF-Nb-Sb-Wo, OF-No-So-Wb⟷OF-Nb-So-Wb, OF-No-Si-Wb⟷OF-Nb-Si-Wb, OF-No-Sb-Wi⟷OF-Nb-Sb-Wi
- IF-No-Si-Wo⟷IF-No-Si-Wb, IF-Ni-Si-Wo⟷IF-Ni-Si-Wb, IF-Ni-So-Wi⟷IF-Ni-Sb-Wi, IF-Ni-So-Wo⟷IF-Ni-Sb-Wo, IF-No-So-Wo⟷IF-No-So-Wb, IF-No-So-Wo⟷IF-No-Sb-Wo, IF-Ni-So-Wo⟷IF-Ni-So-Wb, IF-No-So-Wi⟷IF-No-Sb-Wi
- OF-Nb-So-Wi⟷OF-Ni-So-Wi, OF-Nb-Si-Wo⟷OF-Ni-Si-Wo, OF-Nb-Si-Wi⟷OF-Ni-Si-Wi, OF-Nb-So-Wo⟷OF-Ni-So-Wo
- OF-No-So-Wi⟷OF-No-Sb-Wi, OF-No-So-Wo⟷OF-No-So-Wb, OF-Ni-So-Wo⟷OF-Ni-Sb-Wo, OF-No-Si-Wo⟷OF-No-Si-Wb, OF-No-So-Wo⟷OF-No-Sb-Wo, OF-Ni-Si-Wo⟷OF-Ni-Si-Wb, OF-Ni-So-Wo⟷OF-Ni-So-Wb, OF-Ni-So-Wi⟷OF-Ni-Sb-Wi
- IF-Nb-Si-Wo⟷IF-Nb-Si-Wb, IF-Nb-So-Wo⟷IF-Nb-Sb-Wo, IF-Nb-So-Wo⟷IF-Nb-So-Wb, IF-Nb-So-Wi⟷IF-Nb-Sb-Wi

1.6 Tempering

In order to avoid trapping in deep "energy" (high fitness) basins we employ a tempering procedure. This procedure cyclically raises and lowers the inverse-temperature β in the Metropolis-Hastings acceptance criterion, facilitating the exploration of different areas in the model space because of the increased likelihood of acceptance. ModelExplorer allows for both adaptive and fixed-cycle tempering. Adaptive tempering tracks the change in fitness of the previous models, decreasing β (heating) if the fitness has not changed over several models, and then increasing β (cooling) once a user-defined threshold is met (see below). Fixed-cycle tempering sets a fixed heating and cooling schedule for the duration of the simulation. The tempering procedure is fully customizable and increases the diversity of models found in a simulation. Figure 2 (main text) is an example of the Monte Carlo "energy" trajectory in model space produced in ModelExplorer for a simple symporter system using fixed-cycle tempering. Note that the initial models which are not easily visible at the very left of the graph exhibit poor fitness (positive MC energy), but negative-energy models are quickly found.

1.6.1 Automated tempering

Automated tempering tracks the change in fitness of the previous models, decreasing β (heating) if the fitness has not changed over several models, and then increasing β (cooling) once a user-defined threshold is met. An initial β is set and then checked at fixed Monte Carlo step intervals. At these intervals, the fractional Monte Carlo energy difference from that previous checkpoint is calculated: $E_{MC}^{\text{frac}} = 2\frac{E_{MC}^{\text{new}}-E_{MC}^{\text{old}}}{|E_{MC}^{\text{new}}|-|E_{MC}^{\text{old}}|}$ where E_{MC}^{new} is the current Monte Carlo energy and E_{MC}^{old} is the Monte Carlo energy at the previous checkpoint. If the fractional energy is approximately constant, $|E_{MC}^{frac}| <$ tolerance, β decreases (heats) by a user constrained scale factor. If the fractional energy difference has decreased, β increases (cools) by a user constrained scale factor, subject to a user-defined probability to stay at the current beta, P_{β}^{stay}. If the fractional energy has increased, β decreases (heats) by a user constrained scale factor, subject to a user-defined probability to stay at the current beta, P_{β}^{stay}.

1.6.2 Manual tempering

Manual tempering uses a fixed schedule that adjusts β by a set amount at each Monte Carlo step interval. The user defines the minimum and maximum β (inverse temperature), and also defines how many Monte Carlo steps to remain at the minimum and maximum β, decrease β from maximum to minimum, and to increase β from minimum to maximum. This schedule can then be scaled by a user-defined factor for further sampling optimization. For enhanced selectivity simulations, the default tempering schedule (found empirically) is: 125 MC steps at the minimum β, 100 MC steps increasing β, 1450 MC steps at the maximum β, and 325 MC steps decreasing β.

1.7 Flux calculation

The flux of a given species x, J_x, is calculated by summing the net flows along a user-defined set of transitions for that species. For the ion, this is the set of transitions from an ion-bound state to an ion-inside state (Nb→Ni). The substrate flux is calculated from the set of transitions from a substrate-bound state to a substrate-inside state (Sb→Si). Similarly, decoy flux is calculated from the set of transitions from a decoy-bound state to a decoy-inside state (Wb→Wi). For simplicity, we have removed 'backdoor' transport which would allow a species to be transported in the opposing direction of the conformational state (e.g. OF-Nb→OF-Ni). Due these added constraints, the flux

for a given species is calculated using only the net flows of the (un)binding transitions for that species in the *inward-facing conformation*.
