# Supplementary material for: A systems-biology approach to molecular machines: Exploration of alternative transporter mechanisms
Source: PLoS Comput Biol. 2020 Jul 2;16(7):e1007884. doi: 10.1371/journal.pcbi.1007884 (PMC7331975; doi:10.1371/journal.pcbi.1007884)
Supplement: S2 Text — Details on the clustering procedure, parameters, and the chosen representative models. (PDF) [file pcbi.1007884.s002.pdf]

2 Model clustering and sampling

Simulations were run for transporters in a 'competitive' environment with a decoy. The resulting models were filtered based on a cost (ion to substrate flux ratio) below 10, and selectivity (substrate to decoy ratio) above $10e^{\Delta\Delta G=1}$. Clusters were determined using hierarchical clustering with complete-linkage and the Euclidean distance between the scaled flows of each model. The threshold of 0.65 was determined empirically to produce qualitatively different kinetic pathways. This method produced four separate clusters. Representative models corresponding to each cluster were analyzed in the main text and SI.

The representative models used:

	Run	MC step number
Cluster A model	1	3500
Cluster B model	1	29000
Cluster C model	3	3000
Cluster D model	3	829000
