# Supplementary material for: A systems-biology approach to molecular machines: Exploration of alternative transporter mechanisms
Source: PLoS Comput Biol. 2020 Jul 2;16(7):e1007884. doi: 10.1371/journal.pcbi.1007884 (PMC7331975; doi:10.1371/journal.pcbi.1007884)
Supplement: S1 Table — Table containing the parameter values used for each of the simulations in this study. (PDF) [file pcbi.1007884.s003.pdf]

1 Simulation parameters

1.1 Cotransporter without decoy substrate

	**Symporter**	**Antiporter**
MC steps	1e6	1e6
Random seed	123456	123456
Maximum ΔE for state/transition $[k_B T]$	1.0	1.0
Tempering schedule	Automatic	Automatic
Tempering tolerance	0.3	0.3
β initial $[k_B T]^{-1}$	1e1	1e1
β scale factor	1e3	1e3
P_β^{stay}	0.2	0.2
MC steps to change β	2e2	2e2
$\Delta\mu_{\text{ion}}$ $[k_B T]$	-4	-4
$\Delta\mu_{\text{substrate}}$ $[k_B T]$	+2	-2
Rate prefactor, k_0 $[\text{s}^{-1}]$	1e-3	1e-3
Energy function, E_{MC}	$-J_{\text{substrate}}$	$+J_{\text{substrate}}$

1.2 Cotransporter with decoy substrate

	**Run 1**	**Run 2**	**Run 3**	**Run 4**
MC steps	1e6	1e6	1e6	1e6
Random seed	456789	456789	456789	456789
Maximum ΔE for state/transition $[k_B T]$	1.0	0.5	0.2	1.0
Tempering schedule	Manual	Manual	Manual	Manual
Tempering scale factor	1.0	0.5	1.0	2.0
β_{min} $[k_B T]^{-1}$	1e-100	1e-100	1e-100	1e-100
β_{max} $[k_B T]^{-1}$	1e30	1e30	1e30	1e30
MC steps to change β	1	1	1	1
$\Delta\mu_{\text{ion}}$ $[k_B T]$	-4	-4	-4	-4
$\Delta\mu_{\text{substrate}}$ $[k_B T]$	2	2	2	2
$\Delta\mu_{\text{decoy}}$ $[k_B T]$	2	2	2	2
$\Delta\Delta G$ $[k_B T]$	1	1	1	1
Rate prefactor, k_0 $[\text{s}^{-1}]$	1e-3	1e-3	1e-3	1e-3
Energy function, E_{MC}	$-J_{\text{substrate}}\frac{\lvert J_{\text{substrate}}\rvert+\epsilon}{\lvert J_{\text{decoy}}\rvert+\epsilon}$	...	...	...
Numerical stability constant, ϵ	1e-15	1e-15	1e-15	1e-15
