# Supplementary figures and images for: A systems-biology approach to molecular machines: Exploration of alternative transporter mechanisms
Source: PLoS Comput Biol. 2020 Jul 2;16(7):e1007884. doi: 10.1371/journal.pcbi.1007884 (PMC7331975; doi:10.1371/journal.pcbi.1007884)

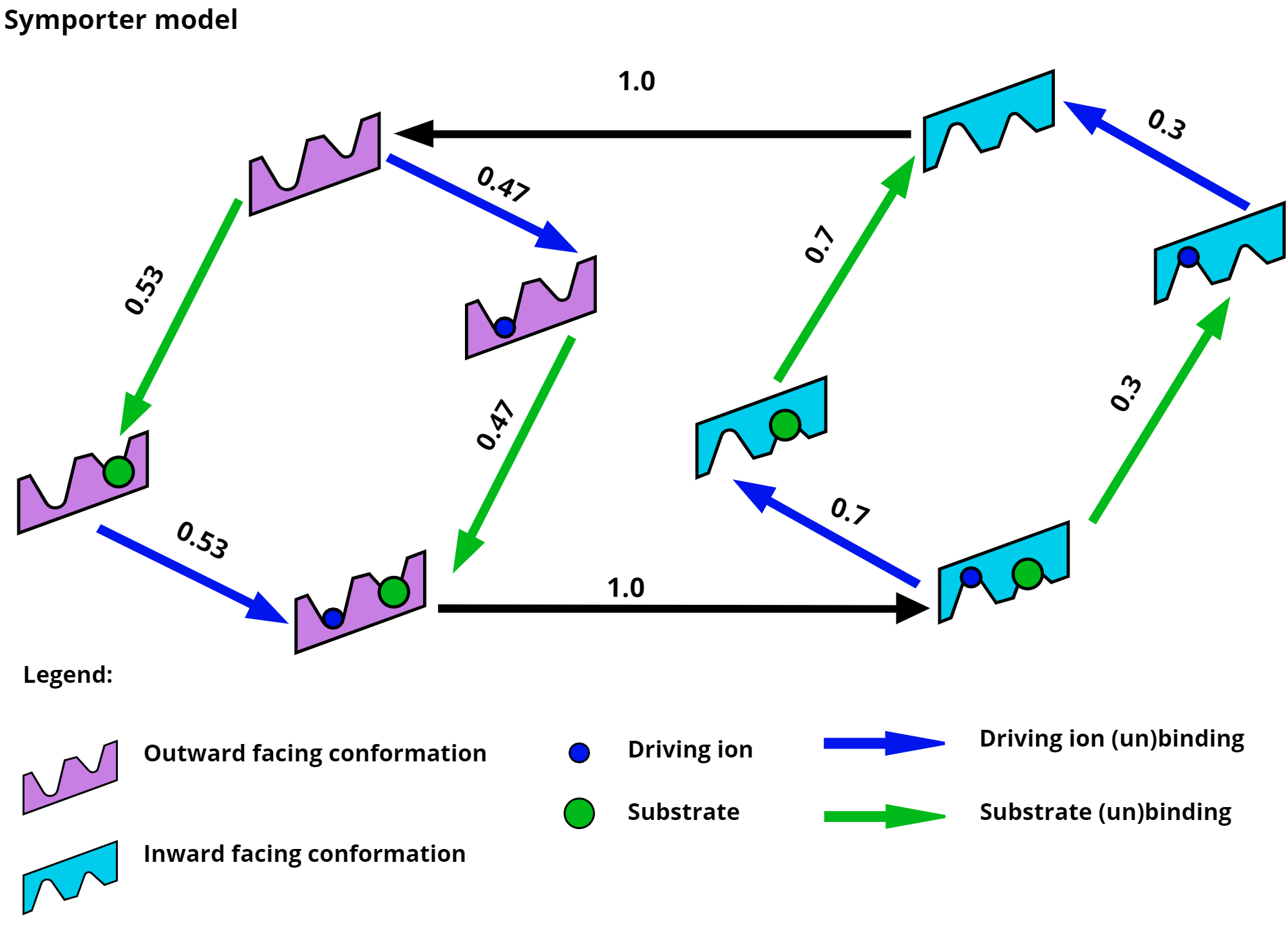

Supplement: S1 Fig — Pathway of a symporter model found at MC step = 1800. This model exhibits a combination of all four ideal symporter pathways which result in the intracellular transport of one substrate per ion. Note that the flows are scaled by the largest flow edge. (TIF) [file pcbi.1007884.s004.tif]

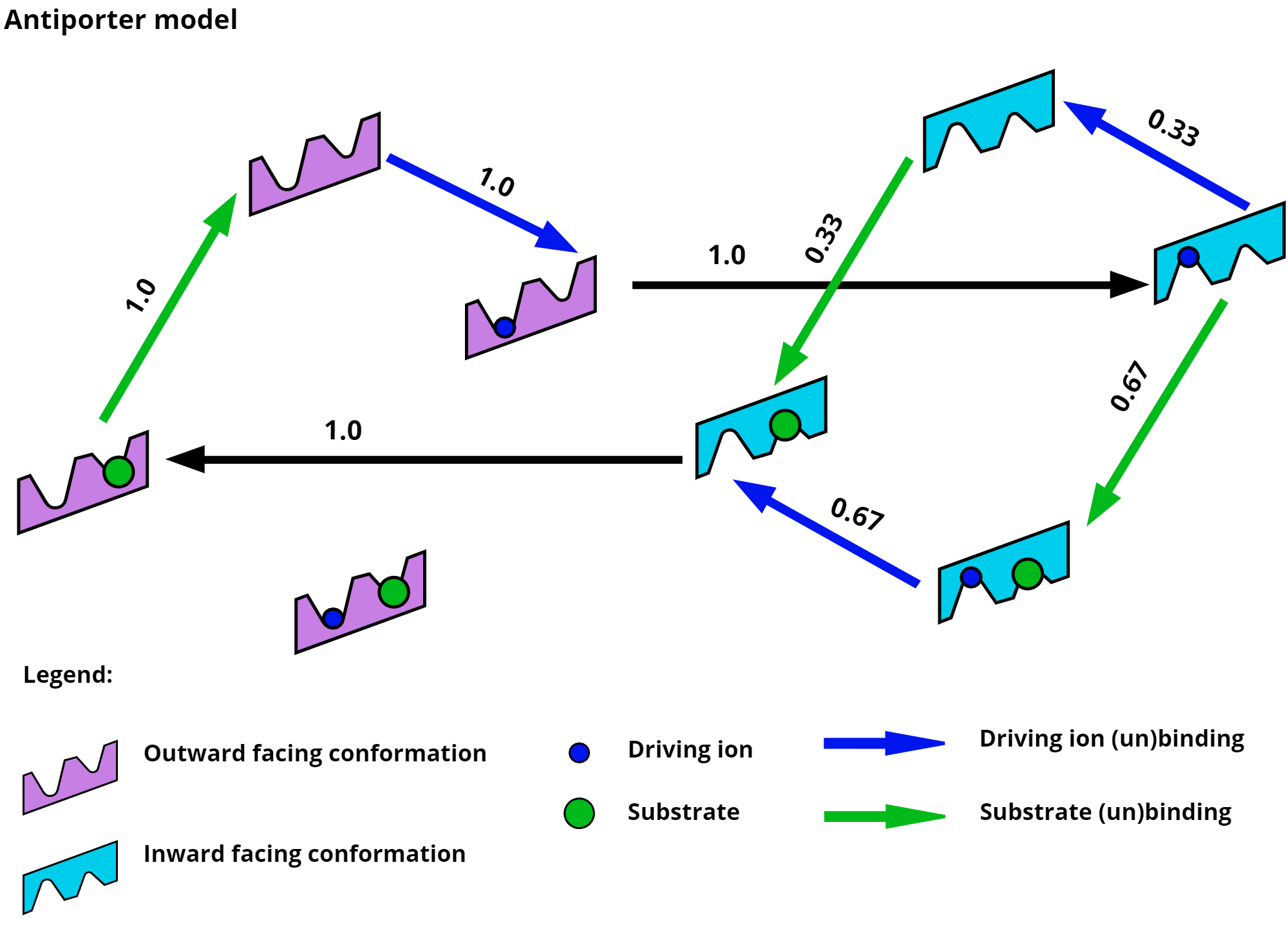

Supplement: S2 Fig — Pathway of an antiporter model found during the antiporter simulation run at MC step = 845000. This model exhibits a combination of two ideal antiporter pathways which result in the extracellular transport of one substrate per ion. Note that the flows are scaled by the largest flow edge. (TIF) [file pcbi.1007884.s005.tif]

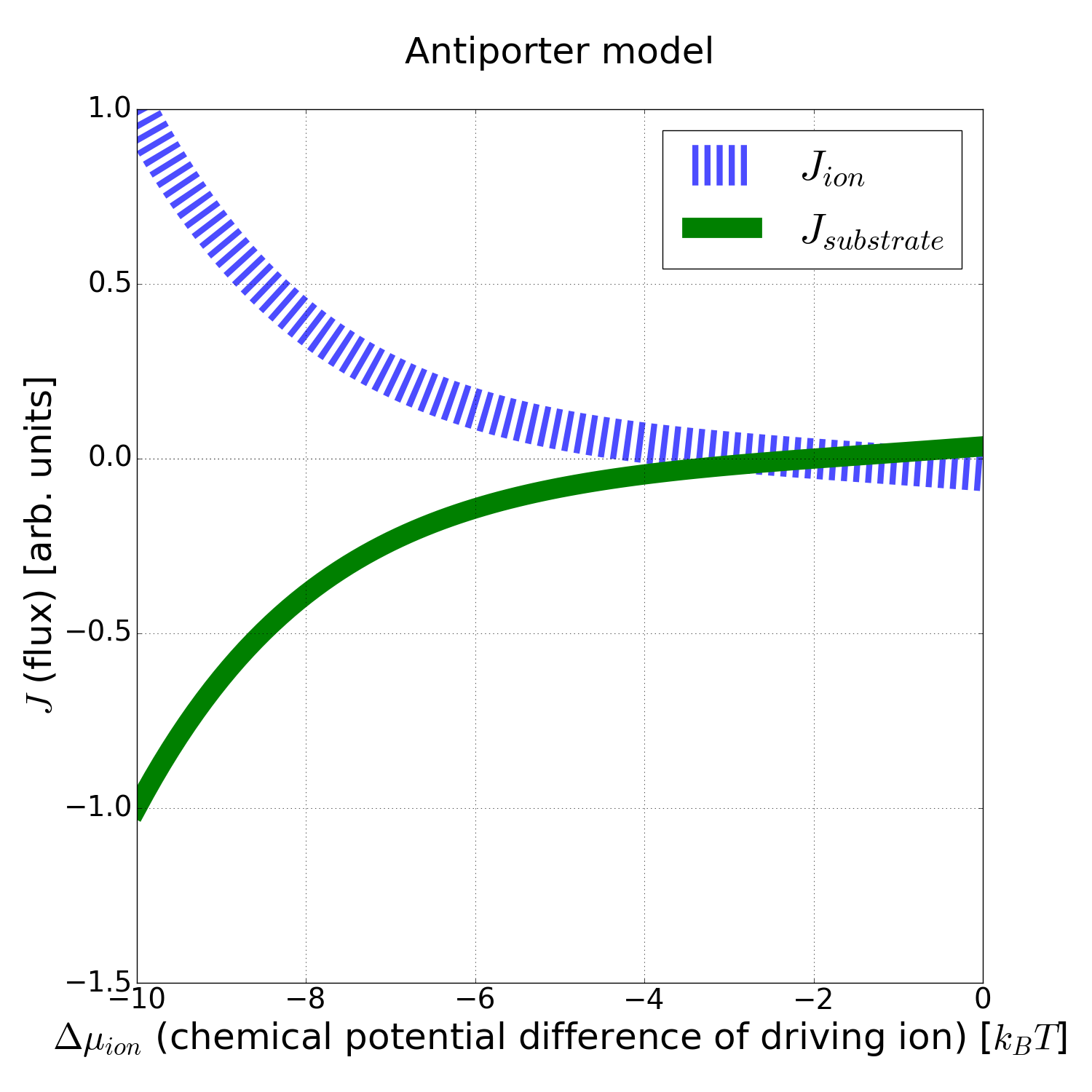

Supplement: S3 Fig — Flux of an antiporter model found during the antiporter simulation run at MC step = 845000, analyzed over a range of ion chemical potential differences. This model exhibits a 1:1 ratio of ion influx to substrate efflux over a wide range of ion chemical potential differences. Note that the fluxes are scaled by the largest flux value. (TIF) [file pcbi.1007884.s006.tif]

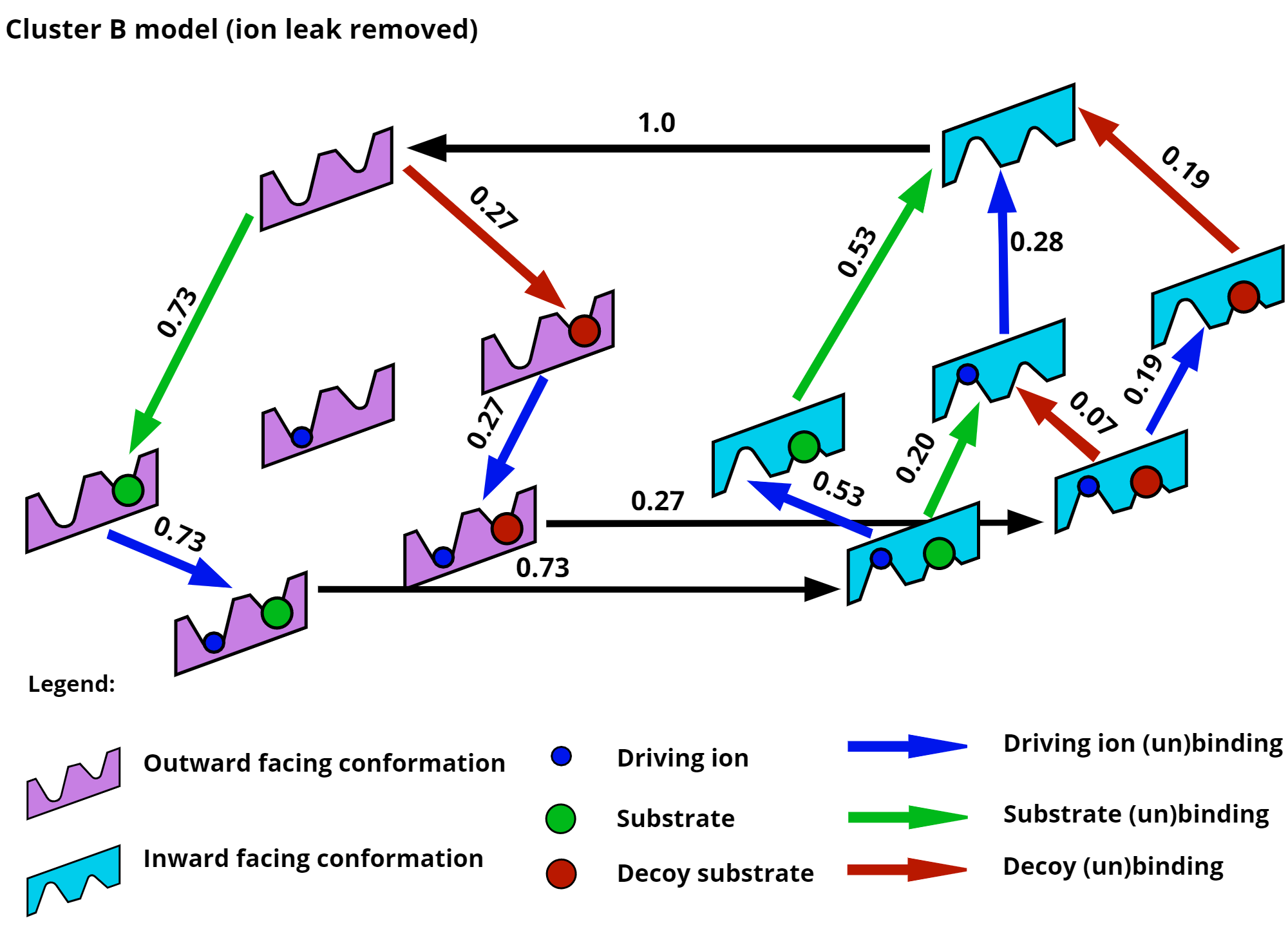

Supplement: S4 Fig — Pathway of the model with enhanced selectivity representing cluster B, with the futile ion cycle removed. The energy barrier between the ion-only bound states in the inward and outward conformations was raised by 100 kBT, effectively shutting off the ion leak. Note the two symmetrical pathways for substrate and decoy transport. The net flows have been scaled by the maximum flow edge. (TIF) [file pcbi.1007884.s007.tif]

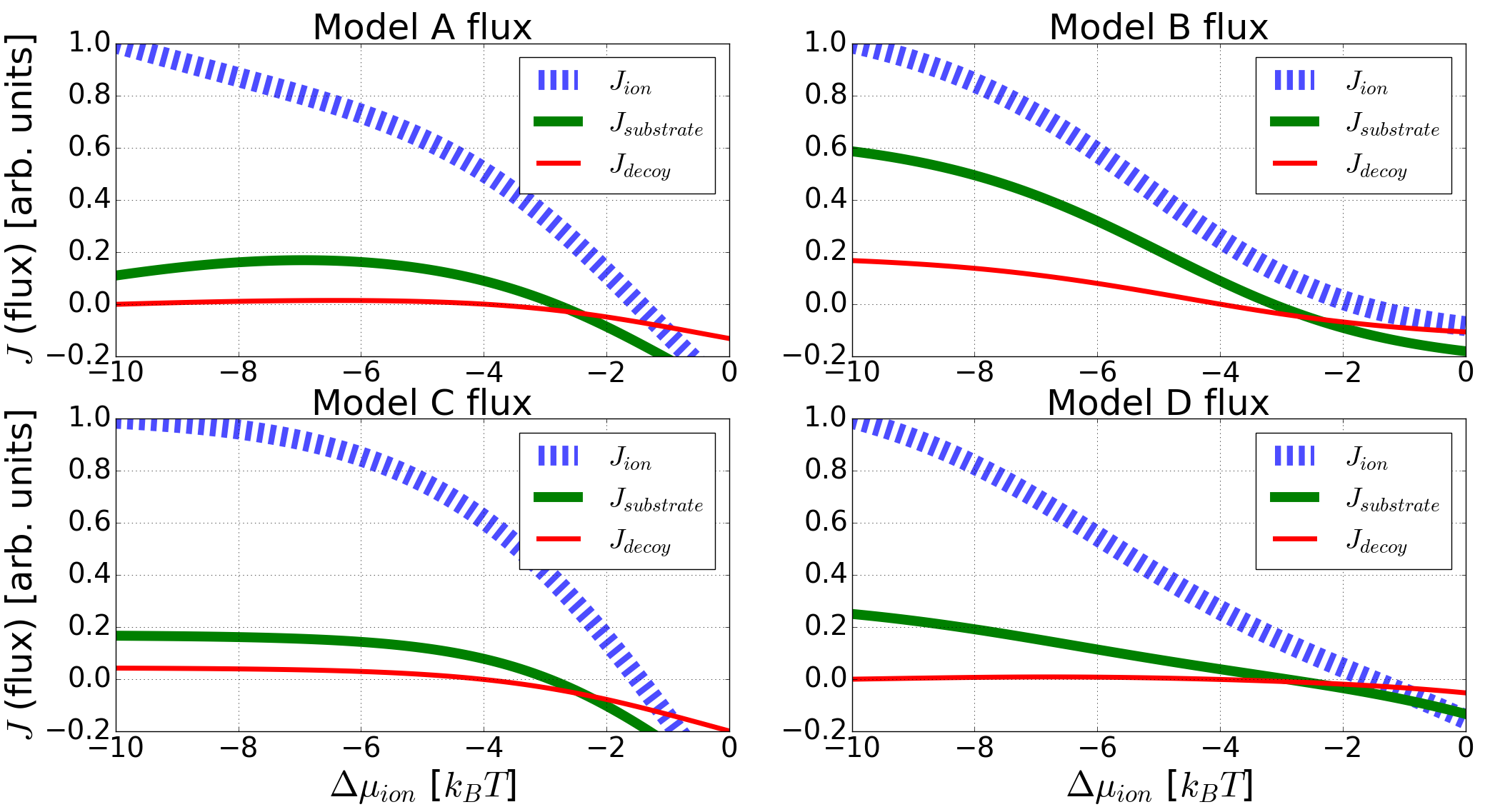

Supplement: S5 Fig — Flux of the representative model for each cluster, scaled by the maximum flux, over a range of ion chemical potential differences. Each model has a narrow regime where the toxin flows down its gradient out of the cell, while the substrate is driven into the cell by the ion. Near the optimized conditions for the simulation, Δμion = −4kBT, these models have negligible decoy flux, resulting in an unbounded substrate to decoy discrimination ratio. (TIF) [file pcbi.1007884.s008.tif]

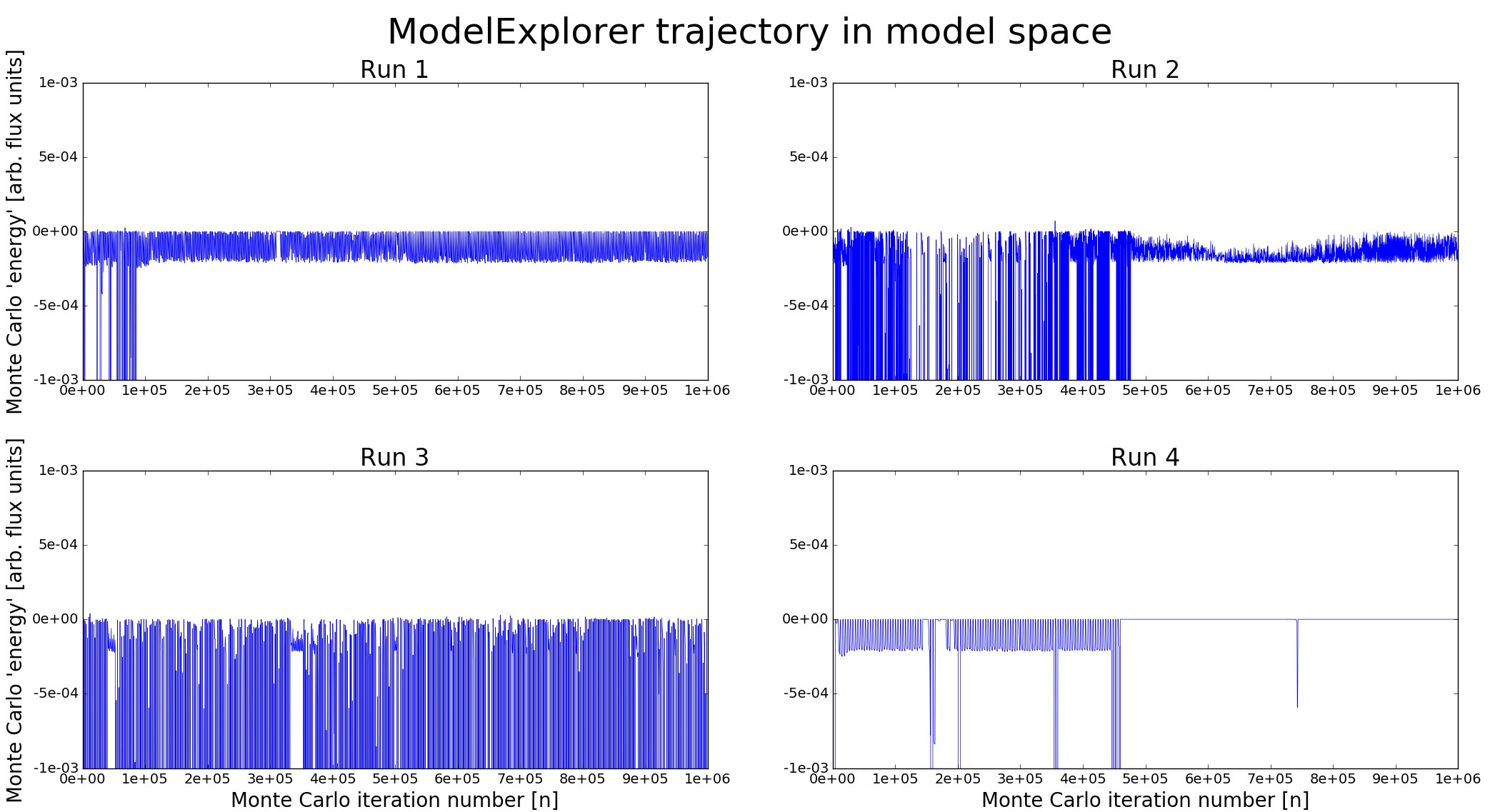

Supplement: S6 Fig — Trajectory in the model space of four different 1e6 Monte Carlo (MC) step simulations at different sampling settings. Simulations were run for transporters in a ‘competitive’ environment with a decoy using the MC energy function: -Jsubstrate|Jsubstrate|+ϵ|Jdecoy|+ϵ where Jsubstrate and Jdecoy are the fluxes of the substrate and decoy respectively, and ϵ = 1e-15. Lower MC energy values denote models that are more fit, by convention. As shown in the figures, the tempering schedule aids in avoiding low energy basins. Note that each point on the trajectory is a kinetic model. (TIF) [file pcbi.1007884.s009.tif]

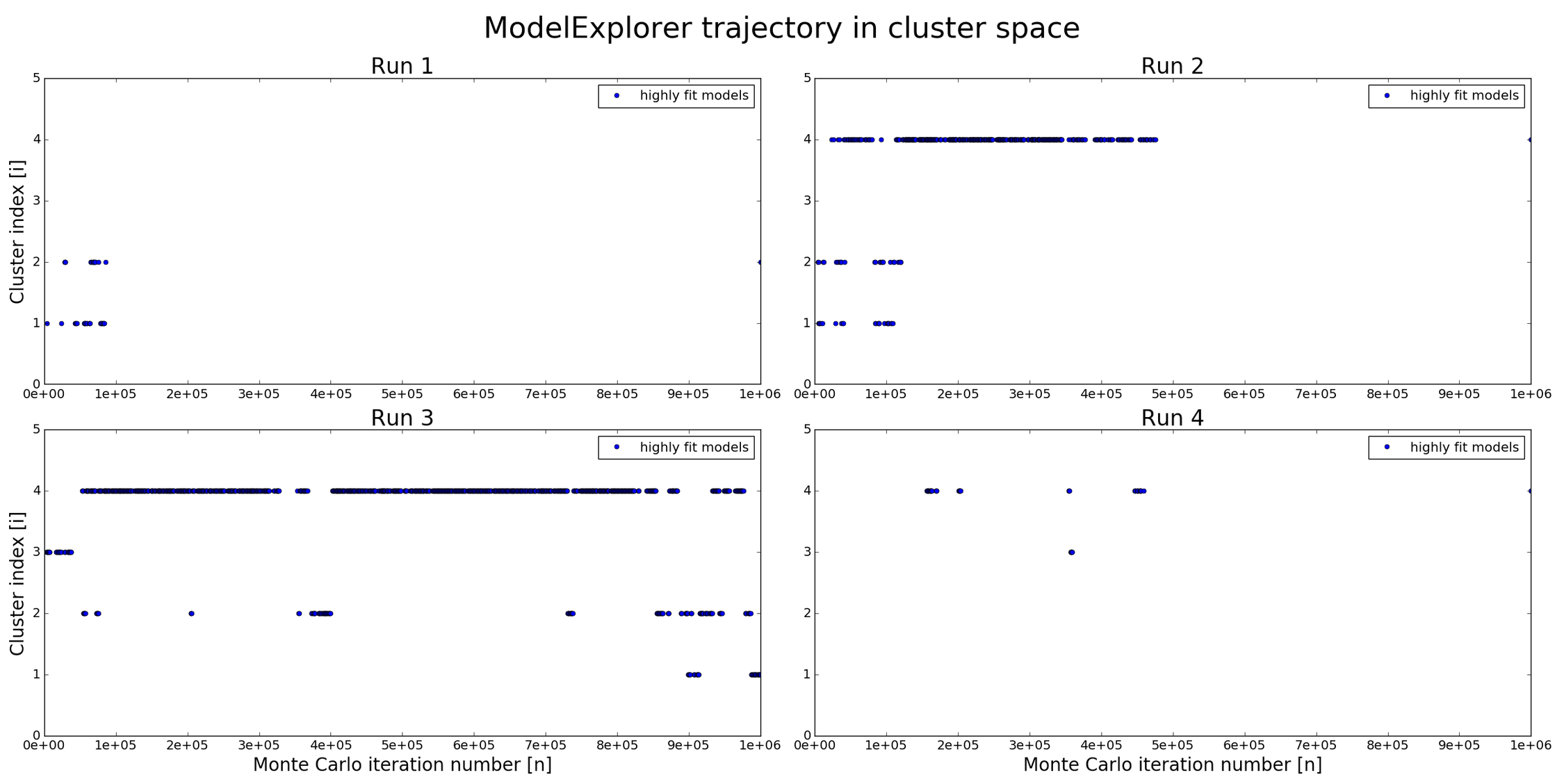

Supplement: S7 Fig — Trajectory in the cluster space of four different 1e6 MC step simulations. Simulations were run for transporters in a ‘competitive’ environment with a decoy. Models were filtered based on a cost (ion to substrate flux ratio) below 10, and selectivity (substrate to decoy ratio) above 10eΔΔG=1. Clusters were determined using hierarchical clustering with complete-linkage and the Euclidean distance between the scaled flows of each model. The threshold of 0.65 was determined empirically to produce qualitatively different kinetic pathways. These graphs indicate that each run only finds a few model classes during the simulation—implying the need for improved sampling methods. Note that in run 4, models meeting the selection criteria (i.e. cost and selectivity) were not found until approximately 1.5e5 MC steps. (TIF) [file pcbi.1007884.s010.tif]

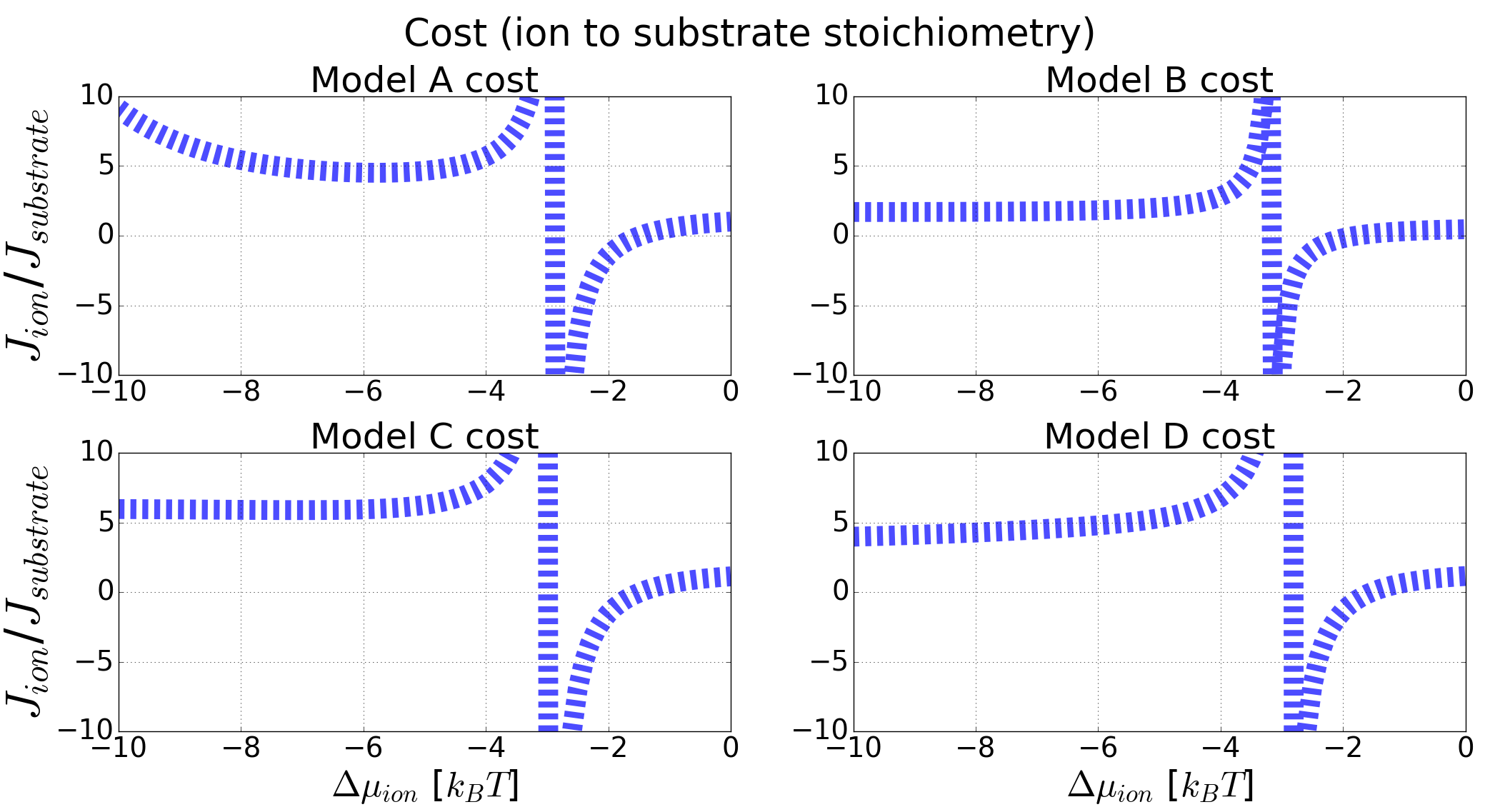

Supplement: S8 Fig — The cost of the representative model for each cluster, over a range of ion chemical potential differences. All of these models exhibit a cost above the ideal 1:1 stoichiometric ratio for a wide range of chemical potential differences. The extra ions transported relative to the substrate suggest a futile ion transport cycle—i.e. an ion leak. Note that the cost was not included as a constraint in the energy function. (TIF) [file pcbi.1007884.s011.tif]

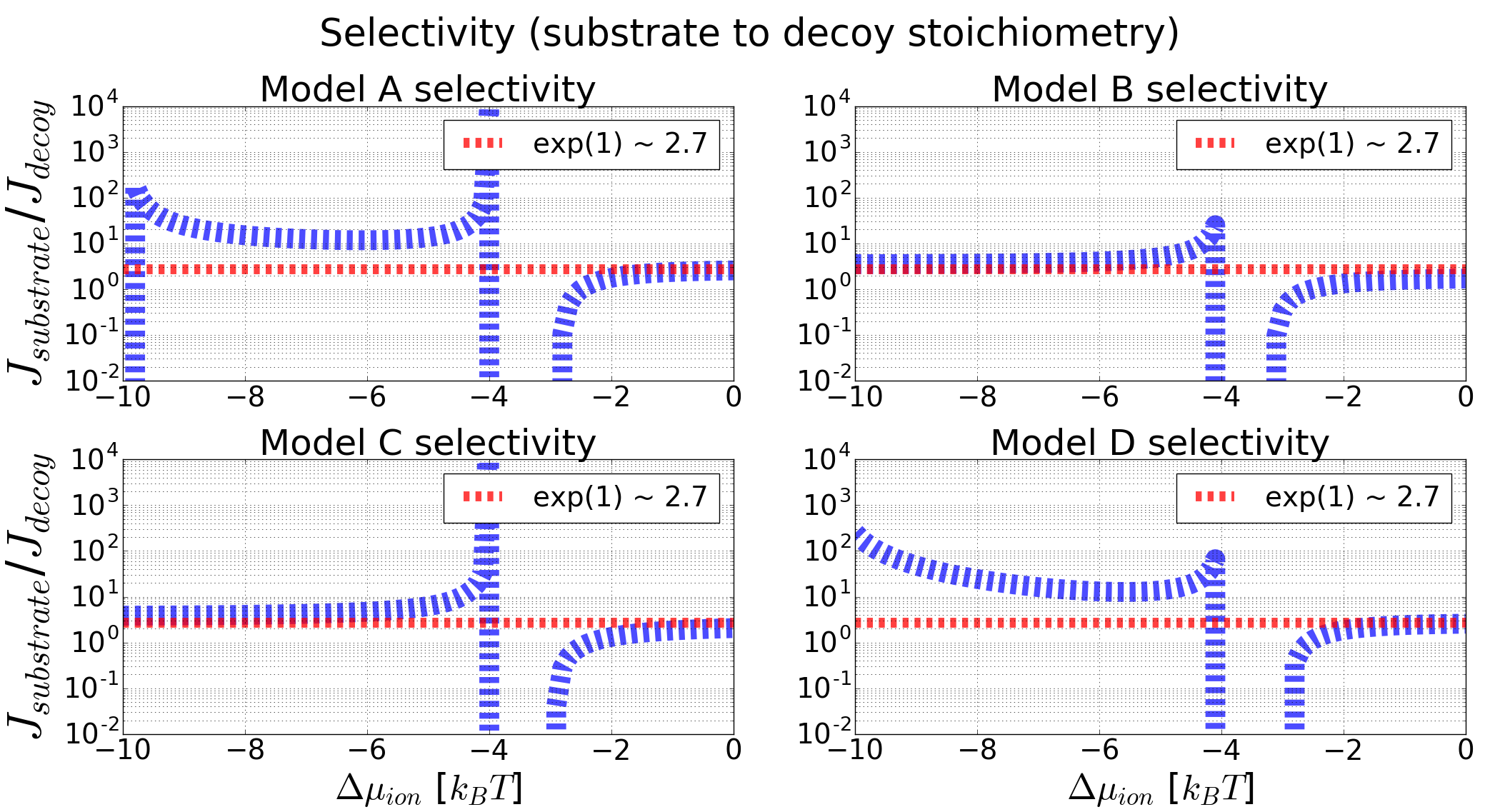

Supplement: S9 Fig — The stoichiometric ratio of the substrate to ion flux (selectivity), over a range of ion chemical potential differences. All the models demonstrate enhanced selectivity over a range of chemical potential differences, and unbounded selectivity at the optimized condition (at Δμion = -4kBT). Models A and D exhibit enhanced discrimination over a wide range of conditions. The expected equilibrium value (eΔΔG=1) is shown as a reference. Note that this stoichiometric ratio was used as a primary constraint in our energy function. (TIF) [file pcbi.1007884.s012.tif]
